# From disease to desire, pleasure to the pill: A qualitative study of adolescent learning about sexual health and sexuality in Chile

**DOI:** 10.1186/s12889-015-2253-9

**Published:** 2015-09-23

**Authors:** Anna K-J Macintyre, Adela Rosa Montero Vega, Mette Sagbakken

**Affiliations:** Department of Community Medicine, Institute of Health and Society, University of Oslo, P.O Box 1130, Blindern, Oslo, 0317 Norway; Centre for Reproductive Medicine and Adolescent Development (CEMERA), Faculty of Medicine, Universidad de Chile, Profesor Zañartu 1030, Independencia, Santiago, Chile; Department of Nursing, Faculty of Health Sciences, Oslo and Akershus University College, Oslo, Norway; National Centre for Minority Health Research (NAKMI), Gullhaugveien 1-3, Oslo, 0484 Norway

**Keywords:** Sexuality, Sexual health, Reproductive health, Sex education, Adolescents, Chile

## Abstract

**Background:**

Sexual and reproductive rights include access to accurate and appropriate information in order to make informed decisions. In the current age of media globalization and Internet, adolescents are exposed to information about sexual health and sexuality from a myriad of sources. The objective of this study was to explore sources of information and adolescent learning about sexual health and sexuality in Santiago, Chile.

**Methods:**

Data collection included four focus group discussions with a total of 24 adolescents 18–19 years old, 20 semi-structured interviews with adolescents 16–19 years old, and seven interviews with key informants working with adolescents. Audio recordings were transcribed verbatim and analysed using content analysis.

**Results:**

The primary sources of sexual health and sexuality information were parents, teachers and friends, whilst secondary sources included health professionals for females and Internet for males. Information provided by the trusted sources of parents, teachers and health professionals tended to focus on biological aspects of sexuality, particularly pregnancy and sexually transmitted infections. Limited emphasis was placed on topics such as love, attraction, pleasure, relationships, abstinence and sexual violence. Information focused primarily on heterosexual relations and reproduction. Adolescents learnt about relationships and sexual acts through friends, partners and, for many males, pornography. Findings indicate a lack of available information on partner communication, setting personal limits, and contraception, including morally neutral and medically correct information about emergency contraception.

**Conclusions:**

This study highlights numerous gaps between adolescent information needs and information provided by parents, teachers and health professionals. The priority these trusted sources place on providing biological information overshadows learning about emotional and relational aspects of sexuality. This biological rationalization of adolescent sexual behaviour neglects the way gender inequality, peer-pressure, coercion, media eroticization and religion influence adolescent sexual decision-making. The heteronormativity of information excludes other sexual orientations and disregards the diverse spectrum of human sexual behaviours. Finally, the limited provision of practical information hinders development of skills necessary for ensuring safe, consensual and pleasurable sexual relations. Trusted adults are encouraged to engage adolescents in critical reflection on a broad range of sexuality topics, dispelling myths, and building knowledge and skills necessary to make informed decisions.

## Background

Adolescence is a time of considerable sexual development, driven by biological and cognitive changes entering puberty, coupled with changes in social expectations and interactions [1]. For both biological and sociocultural reasons, adolescents are at increased risk of poor sexual health outcomes such as sexually transmitted infections (STIs) and unintended pregnancy [2]. Furthermore, adolescence is a time of increased vulnerability to sexual coercion and violence due to lack of experience, social pressure, and the progressive development of self-identity and self-esteem [1]. Sex education is one of the core strategies for achieving improved adolescent sexual health [2, 3].

Desiging effective sex education has been the subject of numerous studies and characteristics of succsessful school sex education programs have been extensively decribed [4, 5]. Many of these studies have shown a positive effect on knowledge of safe sex practices, however knowledge alone may not always be enough to ensure safe sex behaviours. In their literature review, Marston and King [6] point to wider barriers that inhibit safe sex practices, beyond a lack of knowledge. These include poor communication, myths and stigma related to condom use and gender stereotypes inhibiting safe sex practice or partner communication [6]. Thus to be effective, sex education should focus on increasing knowledge, developing skills and challenging sociocultural barriers to safe sex. In addition, sex education programs need to consider the challenges and opportunities presented by the unique sociocultural and political settings where the programs are implemented. In Chile this includes taking into account issues surrounding legal rights (or lack thereof), religion, indigenous worldviews, immigration, societal and cultural norms and gender roles.

Much literature on sex education is focussed on formal education in schools, however studies from settings as diverse as the United States [7–9], Vanuatu [10], Nigeria [11] and Brazil [12] attest to adolescents receiving and actively seeking sexual health information from a range of formal and informal sources simultaneously. Furthermore, the explosion of media globalization with the Internet has opened up new forums for sexual health and sexuality information [13]. Independent of where this information comes from, it may be ambiguous and contradictory, clouded in secrecy and emotional overtones [14], and shaped by the attitudes, ethics and values of the adult world [15].

In the Chilean context, previous quantitative studies name schools [16] and parents [17] as the main sources of sexual health and sexuality information utilized by adolescents. From these studies it remains unclear what information is provided by these sources, how this information is communicated and how adolescents judge the trustworthiness of the information they receive. This current research aims to address this gap in the literature.

This study had the main objective to explore sources of information and adolescent learning about sexual health and sexuality in Chile. This paper reports on four sub-objectives exploring 1) the sources of information Chilean adolescents use to learn about sexual health and sexuality; 2) the content of this information; 3) the techniques used to communicate this information; and 4) the strategies Chilean adolescents adopt to judge the trustworthiness of the information.

### Study context

Chile is a traditionally conservative country, reflected in strong links between religious and political power, conservative "pro-family" legislation and strongly prescribed gender roles [18]. In the years immediately following the return to democracy in 1990, Chile made great advances in civil and political rights, however moves to improve sexual and reproductive rights were slow and inconsistent [15, 18].

From the mid 2000s onwards, considerable legal advances were made. In 2010, a law was passed ensuring the right to sex education, access to information and services for the prevention of adolescent pregnancy, (including emergency contraception) and outlining the legal responsibility of health professionals to report suspected sexual abuse [19]. This final point is of key importance since a 2012 national survey found 7.3 % of school-aged children had experienced some form of sexual abuse, whilst 22.4 % of women surveyed had experienced sexual violence at some point in their lives [20]. In 2012 an anti-discrimination law was passed which included discrimination on the grounds of sexual orientation [21]. Although discrimination based on sexual orientation and/or gender identity still exists, there has been a reduction in reported cases of violence against homosexual, lesbian, bisexual or transgender individuals since the peak of 186 cases in 2011 [22]. Even though advances in sexual and reproductive rights have been made, major issues remain, such as the penalization of induced termination of pregnancy, including therapeutic abortion.

Chile has also seen changes in epidemiological trends in adolescent sexual and reproductive health. Adolescent pregnancy rates peaked in 2008–2009, followed by a downward trend thereafter [23]. In 2012, 14.4 % of all live births (34,900 births) were to adolescent mothers 10–19 years old, down from 16.6 % (40,927 births) in 2008 [23]. Regarding STIs, the most commonly diagnosed infections in Chile are genital warts (caused by the human papilloma virus) and syphilis [24]. The highest number of new notifications for STIs and human immunodeficiency virus (HIV) are in the age group 20–39 years [24, 25]. It is unknown how many of these adults may have been infected during adolescence, especially given that prevention of pregnancy, rather than prevention of STIs has been described as the primary motivation for using condoms by Chilean youth [16, 25].

## Methods

### Study design

This study is based on four months of field research in Santiago, Chile in 2013. Data collection included two pilot interviews with university students, two focus group discussions (FGDs) with a total of 14 high school pupils 18–19 years old, two FGDs with 10 university students 18–19 years old, 20 semi-structured interviews with high school pupils 16–19 years old, and seven semi-structured interviews with key informants working with adolescents. The semi-structured interview method was chosen to collect data on individual experiences, perspectives and opinions, whilst the focus group discussion method was chosen to collect data on overarching themes, as well as group dynamics.

The initial pilot interviews were conducted to practice the interview structure, test the interview questions and tailor the interview guide to the Chilean context. Similarly, the first two FGDs with high school students helped adjust the interview guide through discussion of sources of information previously identified from the literature and encouraging discussion of any new sources the adolescents identified. Furthermore, these discussions provided a valuable opportunity to observe how adolescents discussed the study topic in a group setting.

For individual interviews, a total of 20 adolescents were recruited from three high schools in the municipalities of Independencia, Recoleta and Las Condes. These three high schools were chosen to capture a diversity of youth experiences as the schools differed in size, socioeconomic status, academic rigour, geographic location, religiosity and sex education curriculum.^1^ One school was private and Catholic, whilst the other two were partially subsidised, secular schools. All schools differed in their way of approaching sex education, and even in the same school between different year levels there were significant differences.

Upon completion and preliminary assessment of adolescent interviews, seven semi-structured interviews were held with key informants working with adolescents, including three school psychologists, three health professionals (midwife, paediatrician and gynaecologist) and one Catholic priest. Finally, upon completion of all individual interviews, a further two FGDs were held at a public university to stimulate fruitful discussions on the preliminary findings.

All interviews and discussions were conducted/moderated in Spanish by the first author with the support of a local research assistant to resolve language misunderstandings and explain culturally specific terms.

### Ethical considerations

Verbal and written consent/assent was collected from all participants after talking through the intentions of the study, structure of the discussion/interview and confidentiality. Written parental consent was required for participants under 18 years of age.

There was no direct benefit to participation and the potential harm was assessed to be low, as the aim was to study adolescent *sources of information* and *learning* about sexual health and sexuality, and not actual sexual *experience.* There was no financial incentive for participation, however snacks and drinks were provided.

This study was approved by the Board of Ethics at the Faculty of Medicine, University of Chile, Santiago and the Norwegian Social Science Data Service.

### Sampling and recruitment

Conducting research within the constraints of the high school setting meant that sampling and recruitment needed to be both focused and flexible, juggling the busy schedules of the school psychologists aiding recruitment, the pupils and the interview team (first author and research assistant). In response to these challenges and to ensure a gender balanced sample with maximum variation, a range of sampling techniques were utilized. This included maximum variation sampling, purposeful sampling of adolescent parents and purposeful random sampling (when more adolescents volunteered than time constraints would allow for interviewing) [26]. For FGDs, homogenous sampling was the primary approach used, however poor participant turn out on each day of the high school FGDs mean that opportunistic sampling was necessary [26].

The project was presented by the first author to entire classrooms of high school or university students or to a selection eligible pupils (for example all pupils present over the age of 18 in the high schools). 11 adolescents over 18 years of age volunteered and seven of these were interviewed. Those volunteers under 18 years of age were given an informed consent form to take home to their parents. In total 18 out of the 39 volunteers (46 %) returned parental consent forms and 13 of these were interviewed.

For sampling key informants, information from adolescent interviews and FGDs highlighted the importance of schoolteachers and health professionals in provision of information. The influence of religion on sexual health discourse in Chile was also discussed, therefore a representative of the Catholic Church with extensive experience working with adolescents was recruited. These participants were all contacted directly by the first author and invited to participate.

### Interviews

The final sample included 10 females and 10 males, three of whom were adolescent parents. The interviews opened with a general request for the participant to introduce themselves, before being asked to describe in their own words what they understood by the terms *sexual health, sex education* and *sexuality.* This was in order to ensure intersubjectivity: that the researcher and participant had a common understanding of the core terms used throughout the interview. Participant responses to interview questions were understood in relation to their unique understanding of these terms and probes were used to integrate elements of the interviewer's understanding.

The core of the interview included questions and probes on each source of information identified from the literature, pilot interviews and FGDs: family, school, friends, Internet, health professionals, television, films, advertisements, radio and religion. It was neither the aim nor was it possible to ask all questions in all interviews. Some adolescents required a more exploratory approach with few open questions, whilst other adolescents required a more descriptive approach with specific questions and extensive probing.

For key informant interviews, separate interview guides were developed for each group after pre-analysis of adolescent interviews. These guides included exploratory questions as well as examples of anecdotes or relevant themes from adolescent interviews and FGDs brought up for discussion.

Interviews were conducted in private offices in the schools and key informant interviews were held in the offices of each participant. All interviews were audio recorded and lasted 45–75 min.

### Focus group discussions (FGDs)

Four gender separated FGDs were conducted with a total of 24 adolescents (12 female, 12 male) 18–19 years old. For the first two FGDs in the high school the discussion opened with an ‘ice breaker’ activity whereby all participants were asked to write anonymously a list of all the words they knew related to *sex*, *sexual health* and *sexuality*. After discussion of a selection of these words, open questions were posed about different sources of information and how they believed Chilean adolescents utilized these to learn about sexual health and sexuality. There was also room for probing on emergent themes.

For the final two FGDs a new thematic guide was developed with an ‘ice breaker’ activity where the participants were presented with the lists of words created by participants in the first focus groups and asked to comment on these. Following on, the guide included a number of themes or anecdotes from adolescent interviews that were interesting, surprising or particularly noteworthy. Again, there was room for spontaneous probing on emerging themes.

The FGDs were held in an empty school library and a university conference room. The discussions were audio recorded and lasted 50–80 min.

### Data analysis

Data analysis started upon entering the field through debriefing, journaling, prompt verbatim transcription of interviews/discussions and eight formal pre-analysis discussions in the interview team. All interviews and discussions were transcribed verbatim in the field by the first author and research assistant. Afterwards a selection of transcripts were interchanged between the two and crosschecked for accuracy. During the pre-analysis sessions transcripts were reviewed and recurrent or emerging themes were recorded. Throughout the data collection process the interview guide was continuously adjusted and through pre-analysis discussions it was possible to determine a point of adequate saturation of data in relation to the research objectives.

Upon completion of fieldwork, the formal data analysis drew on Taylor-Powell and Renner's steps in content analysis [27]. Initially, in order to form an understanding of the data set as a whole, all interviews were listened through and transcripts were reviewed. Thereafter, the data was manually coded "descriptively" with the creation of 36 descriptive codes by source of information stemming from the research objectives and interview guides (for example *home: sexual orientation*). Following on, the data was coded analytically, and through a flexible process of code expansion and deletion, categories developed based on the content, rather than source of information (for example *homosexuality: biological explanation*). These categories were recorded in colour-coded tables with examples the data, translated by the first author from Spanish to English, and checked by a native Spanish speaker. In the final step the data was analysed within the context of relevant theories and previous empirical research, leading to the development of three major themes (two of which will be discussed in this paper).

## Results

The primary sources of information utilized by Chilean adolescents in this sample are family, school and friends. There were gender differences in the utilization of family as a source, with females more likely to talk with one of their parents than males. Many adolescents talked with friends or partners about topics related to sexual health and sexuality. Although these conversations were seen as important, there was often resistance to the idea of friends and partners being sources of information in the formal sense.

There were visible gender differences in the use of secondary sources, with females using health care services as a source of information whilst males were more likely to utilize Internet. Alternative media sources such as television, films, advertisements and radio were only sporadically mentioned, therefore they will not be reported on further in this paper. Similarly religion, or specifically representatives of Catholic or Evangelical Churches were not seen as sources of information per se, however religion was discussed in relation to other sources such as schools.

### Most trusted sources of information

The most highly trusted sources of information were teachers, parents and healthcare professionals. Reasons for this included the innate belief that these adults wished them well, the life experience that these adults had and the length of time the health professionals and teachers had spent studying for their careers. Triangulation of information between these three trusted sources increased trustworthiness.

#### Biological teachings

All the adolescents interviewed received information about the biological aspects of sexuality from school and/or their parents. This included the topics of pregnancy, sexually transmitted infections (STIs) and contraception (most commonly oral or injectable contraceptives and condoms). In schools these topics were most often covered in biology classes, with an academic focus, through powerpoint presentations with limited pupil participation. If covered in orientation classes, sexuality was often taught alongside the topics of drugs and alcohol, with a focus on risk.

Many participants described their feelings of fear and/or disgust at being shown pictures of infected genitals during these sex education classes. Even though they were subsequently informed to use condoms to prevent STIs, only one participant witnessed a practical demonstration of how to put a condom on a broom. One participant shared his opinion of the absence of practical teachings of how to use a condom:

*This is what is also missing in school […] In school they don’t teach you how to put a condom on. So obviously, you could arrive to the act and it doesn’t help you to know all the theory if you don’t know the practice. (Male, 16 years, Interview)*

Formal school education focused on heterosexual relations and reproduction, therefore the only opportunity adolescents had to learn about the broad spectrum of sexual acts or other types of unanswered questions was through anonymous question sessions. During these sessions, adolescents posed questions about a range of topics moving far beyond coitus, for example incorporating topics of oral/anal sex and masturbation. One participant in the Catholic school described an example from such an anonymous question session:

*We were so young that the questions were like… "what does semen taste like". (Female, 16 years, Interview)*

Similarly, two key informants described holding anonymous question sessions and being asked about a range of topics including bestiality and their own sexual experiences.

Parents were another source of biological information with 17 adolescents having received information from their parents about pregnancy, STIs and/or contraception. Most commonly these conversations were triggered by a specific event such as an upcoming school camp, upon request from the school, when starting a romantic relationship or in the case of many females, with their first menstruation. One participant described her experience:

*I started dating and then came the massive attack from my parents about adolescent pregnancy and things like that. […] it was like they assumed that I was no longer a little girl, I was now big and they needed to start informing me. (Female, 19 years, FGD, University)*

Parents commonly provided non-specific messages to *"protect yourself"* without explicit information on how to do this. Conversations were either one off experiences of *"the talk",* or the start of ongoing communication. These triggered conversations were most often experienced as embarrassing for the adolescents, however those with subsequent ongoing communication described increasing trust and diminishing embarrassment over time.

In total four participants stated that their parents had themselves been adolescent parents, therefore communication was triggered by a fear that they would repeat the same scenario as their parent. This is exemplified in comments made two by female participants:

*My mother had me at 18, so she always had a fear, the whole family had this fear that I would repeat my mother's experience. (Female, 19 years, FGD, University)*

*Ever since I was very young she made me scared about birth, about the pain and all these things so that I would not fall pregnant. (Female, 19 years, FGD, University)*

Thus, similar to the school setting, fear was also a tool used by mothers in communicating to daughters about sexual health.

Midwives and gynaecologists also provided reproductive and contraceptive information for females who attended health centres with their mothers after their first menstruation, or when starting a romantic relationship. Only two adolescent males had accompanied their partners to see a gynaecologist, however one health professional described a new trend in both public and private health sectors with more males accompanying their partners for contraceptive advice*.*

#### Emotional and relational teachings

Compared to the biological teachings, few adolescents received information about the more emotional and relational aspects of sexuality, falling under the Spanish umbrella term *afectividad*. In this study, adolescents and key informants described *afectividad* loosely as encompassing themes such as love, romance, attraction, desire, pleasure, affection, caring, self-esteem and communication.

When probed about *afectividad*, two male participants associated this topic exclusively to religion. Unsurprisingly therefore, the Catholic priest interviewed discussed at length the importance of sexual relations being within the context of a committed, loving relationship. If topics related to *afectividad* were taught in schools, this was frequently during religion classes*.* One female participant described how, unlike secular schools, religious schools automatically assumed it was their role to teach adolescents about *afectividad.* Another female described how she had learnt about *afectividad* in religion classes, but that as she got older the focus of her school teaching changed:

*They started saying, I don't know, that "love is not anything magical, instead it is just biological reactions" […] like they tried to remove all of the magic. (Female, 19 years, FGD, University)*

Thus a form of biological reductionism converted the *magic* of love to a purely biological process of chemical reactions, separate from emotions. This separation of the biological and emotional/relational aspects of sexuality in mainstream teaching was critiqued by a psychologist whom stressed the importance of integrating psychosocial elements of sexuality including love, when teaching sex education:

*Often I also tell them [the pupils] that one of the risks of sexuality at such a young age, is not just pregnancy and sexually transmitted infections, it is that you fall so deeply in love, that you fall into a depression. (Health professional)*

Key informants who taught sex education gave a number of reasons for why educators focus on biological sexuality, stating it was a reflection of narrow educational and public health agendas in Chile and that biology was easier to teach than *afectividad*. Furthermore, they stated that to teach about sexuality in depth, educators (whether they be teachers, parents or health professionals) needed to acknowledge their own myths regarding their sexuality. Both adolescents and key informants described self-confidence and trust as prerequisites for communicating effectively about sexual health and sexuality.

Love and affection were not spontaneously mentioned by any participant, however when probed, six participants had talked about these topics at home. From home these messages were either that love and sex were two separate concepts or that it was necessary to have love before engaging in sexual relations. One participant stated that parents were those responsible for teaching about values and love to their children, letting schools and health professionals take responsibility for teaching biological sexual health. He described what he had learnt from home:

*When I was young they talked a lot to me about sex being the most beautiful thing that exists in life and that it is a form of sharing love. (Male, 17 years, Interview)*

This positive message of love and sex contrasts to the direct association of sex to risky behaviours such as consuming drugs and alcohol, a common element of school sex education programs.

#### Sexual pleasure and masturbation

None of the participants had learnt about the topic of sexual pleasure in formal sex education and all four key informants that actively worked with school sex education discussed reasons for this omission. The main reason was the belief that talking about pleasure counteracted the aims of sex education; namely to delay sexual debut and reduce adolescent pregnancies. One psychologist stated her opinion that teaching on the topic of pleasure was a challenge, but not impossible, if approached from the angle of emotions. One adolescent shared her opinion of teaching on pleasure:

*I think that if they cover the topic of pleasure, they have to cover it together with the diseases, always subtly, but each one in their place. […] one can’t just cover the diseases to make them scared. (Female, 19 years, FGD, University)*

The only element of sexual pleasure which was incorporated into formal sex education was male masturbation. Four males recounted how school psychologists had talked of masturbation as a natural part of male sexual development. One male recounted his experience:

*The professors tell you that " for you guys it is normal". (Male, 16 years, Interview)*

Even when the topic of male masturbation was broached in the school setting, none of the participants recalled any messages related to female masturbation. One participant critiqued this:

*I remember very strongly in school the taboo topic of knowing oneself, […] It has always surprised me, like, “why can't women touch themselves?” (Female, 19 years, FGD, University)*

This gendered taboo of masturbation was universally reflected in the adolescent interviews.

#### Sexual diversity

The topic of sexual orientation was also often absent in the formal education and one key informant explained why:

*One approaches what one sees, approaches sexuality from the point of view of prevention of adolescent pregnancy, which in its essence is what scares us adults. (Health professional)*

Thus again, the education and public health agendas limit the breadth of teaching on sexuality. This same health professional also said that sexual orientation was often omitted in schools since many people in Chile still viewed sexual diversity as a question of core social values.

If discussed in schools, the topic of sexual diversity was commonly taught in one of two ways. Firstly, five students were taught about respect and not discriminating against someone on the basis of their sexual orientation and in one school there was a student debate about social and legal rights of homosexual people in Chile. All three school psychologists dismissed the idea of sexual orientation being a cause of bullying in their schools, however one participant described discrimination of a classmate based on suspected homosexual orientation and female FGD participants described a negative attitude towards sexual diversity in their school with peers pointing out students believed to be gay or lesbian.

The second perspective was a biological explanation of sexual diversity. Four participants described learning in biology classes that homosexuality was the result of a hormonal imbalance. For example one female described how her teacher had explained why homosexuality occurred:

*Sometimes they had lots of male hormones or female hormones and that is why it happened. (Female, 17 years, Interview)*

Sexual orientation was sporadically discussed in the home setting, often in reaction to issues surrounding sexual diversity in the media or through comments on the increased visibility of same-sex couples on the streets of Santiago. These discussions focused primarily on personal opinions supporting or rejecting sexual diversity, or the importance of not discriminating.

Two health professionals and one school psychologist described referring adolescents suspected of being homosexual, lesbian or bisexual to clinical psychologists and/or psychiatrists for further follow-up. One health professional described her role supporting a homosexual, lesbian or bisexual young person:

*Explaining to him that it [homosexual/lesbian/bisexual orientation] […] could be something transitory or something definite but he has to work on it, because when he is ready to reveal his sexual orientation, he faces a society that is still not prepared. (Health professional)*

On the one hand, these teachers and health professionals medicalized non-heterosexual sexual orientations as a conditions in need of specialist follow-up, whilst on the other hand, referral opened up a supportive space for counselling adolescents who may receive limited support in their home or school/peer settings.

#### Abortion and emergency contraception

The topic of abortion was rarely discussed in the school setting, the exception being the Catholic school where five participants described a student debate on decriminalization of therapeutic abortion. Compared to other participants, these adolescents talked about abortion with fluidity and reflectiveness.

Emergency contraception was scarcely covered in schools and no participant recalled having received information on the biological functioning of the pill or how to access it. One adolescent and one psychologist stated their opinion that teaching about emergency contraception would encourage unprotected sex. If covered, emergency contraception was discussed from a moral standpoint, focusing on responsibility and blame for unsafe sex. One male participant described the message he received in religion classes:

*We need to care for our bodies and avoid having a sexual relation where the female could fall pregnant, instead of just coming and taking the pill, like taking the easy way out. (Male, 16 years, Interview)*

Although a number of female adolescents had discussed contraception with their mothers, few had talked about emergency contraception. One female participant described the message her mother had given her and her sister:

*My mother says that, if one day someone rapes us, it would be the only time when she would accompany us to take it [emergency contraception]. (Female, 16 years, Interview)*

Thus, the moral aspect of taking emergency contraception was discussed in both the school and home settings with a strong discourse of blame and responsibility for having unprotected sex.

#### Sexual abstinence and defining sex

Surprisingly, the topic of sexual abstinence was almost absent in adolescent discourse. Only one participant, an adolescent mother, recalled learning in school that *"abstinence is the best contraception" (Female, 18 years, Interview).* The idea that Chile was going through a generational change in relation to abstinence was common, with one female stating that abstinence was *"no longer relevant for Chilean society" (Female, 18 years, Interview).* A number of adolescents, particularly male FGD participants, laughed at the concept of abstinence. In response to this observation, one key informant shared her opinion that it was important that educators included the topic of sexual abstinence as some adolescents still saw it as a valid option.

School teaching on sexual abstinence was criticised by male participants from the university FGD, who described a double standard whereby youth followed the teaching to abstain from vaginal sex, instead having unprotected oral or anal sex:

*For example, they say “let’s abstain and do it orally”, and in the end they still catch AIDS or some other thing. (Male, 19 years, FGD, University)*

*You still catch other things, […] like abstinence “no I can’t”, “ok, from behind then” (anal sex). But that still won’t do. (Male, 19 years, FGD, University)*

This highlights the ineffectiveness of teaching on sexual abstinence without first understanding how young people define sex, virginity and abstinence. It also underlines the limitations of safe sex messages that focus primarily on prevention of pregnancy rather than prevention of STIs.

Only one evangelical participant discussed abstinence at home, with female friends and partners. A further three participants with religious parents had talked about abstinence before marriage at home, however in all cases, abstinence was only ever referred to as an ideal, rather than a strict rule.

#### Sexual violence, limits and negotiation

When it comes to sexual violence, none of the adolescents in this study described being taught in schools about setting personal limits, issues of consent or partner violence. A school psychologist critiqued this by quoting a Chilean saying:

*I think that it is serious that it [violence in relationships] is not talked about. It is considered part of our culture. “The one who loves you, beats you”. (Psychologist)*

Two female participants recounted being warned at home about the risk of kidnapping and random sexual violence, however only one participant described how her mother told her to set limits with a partner to avoid verbal, physical or sexual abuse.

No participant described having received guidance on knowing when one is ready for sex. Similarly, none of the participants reported having learnt skills for communicating with a partner about the timing of sex and negotiating condom use.

### Talking about sexuality in the informal peer network

Participants also described conversations with friends and a partner (boyfriend or girlfriend) about a variety of sexuality issues. Friends and partners encouraged varied levels of trust, some participants describing increased trust if the friendship had evolved over a long time. However, there was also scepticism about where these friends had found their information.

Friends occasionally broached the topics of contraception or testing for STIs, usually with the purpose of giving safe sex advice. Notably, the three adolescent parents interviewed saw themselves as trustworthy sources of information as they had first hand experience of how tough it was to become parents so young. One of these mothers described her role:

*I always tell them, "please protect yourselves. It's wonderful to have a baby, but, at our age… because it's so early, you're still in school, entering university…" so I'm always like the mother of the group. (Female, 18 years, Interview)*

It was common for participants to talk with same sex friends about various sexual acts. One female participant described a lunchtime conversation with a sexually experienced classmate:

*We asked her everything, how did she do it, why did she do it, how did it start, […] we had so many questions, since we haven't done it yet, we're virgins, so we wanted to know everything. (Female, 17 years, Interview)*

Conversations with same sex peers could also take the form of social pressure to engage in sexual acts. One male described a joke made about a fellow classmate who had not had sex with his girlfriend after two years together, asking *"when are you going to become a man?" (Male, 18 years, Interview).* This example reinforces the gender stereotype that having sex defines *"being a man".* One psychologist described building self-esteem as fundamental part of sex education so that adolescents do not to submit to this kind of social pressure.

Although communication between male and female friends about sex was uncommon, sexual health topics were often discussed with a partner. All participants said it was necessary to discuss contraception with a partner and ten participants recounted previous conversations with a partner. One male described how he felt talking to his partner about contraception:

*I felt… that I was doing the right thing. […] I felt responsible talking about this. (Male, 16 years, Interview)*

It was evident that the participant felt mature in this situation and confident in sharing responsibility for sexual health choices with his girlfriend. This stands in stark contrast to one adolescent mother's response when asked if she had talked with her partner about sexual health or sexuality before her pregnancy:

*P: He told me that I should go to protect myself, and just when I went [to a healthcare clinic] to protect myself, I got pregnant […]*

*I: And did you ever talk about the topic of condoms?*

*P: No, never. (Female, 19 years, Interview)*

This quote illustrates a traditional gendered responsibility for contraception which one health professional described as less and less common nowadays. She observed of a cultural shift with newer generations of young females expecting their partners to accompany them to seek contraceptive advice and demanding shared responsibility for contraception.

When probing adolescents on topics that they thought should be discussed with a partner, 16 participants described the importance of discussing contraception and protection from STIs. Four males and no females spontaneously included pleasure as a topic for discussion.

### Information on the Internet and attitudes towards pornography

Internet was rarely referred to as a source of sexual health information in a formal sense. A number of participants had utilized Internet for school projects, however these participants were sceptical to the truthfulness of the information. All participants who used Internet described different triangulation techniques for assessing critically the trustworthiness of information, often prompted by a trusted adult advising them to be critical of certain websites. These techniques included crosschecking information on various websites to assess the congruency of the information, or double-checking the information found on the Internet with a trusted adult or friend.

Alongside asking sexually experienced friends, searching on the Internet and specifically watching pornography, were described as ways of learning about different sexual acts. When asking about the acceptability of pornography, there was a universal gender difference in the responses. Males described viewing pornography as normal, although potentially only acceptable up to a certain age, whilst females regarded it as taboo. One participant named pornography as a causal factor in adolescent pregnancy:

*There's a misinformation. Because of this I think that sometimes youth, younger than me, have babies […] Because basically in a pornographic video you see people having sexual relations, right? They don’t show you how to put a condom on. (Male, 16 years, Interview)*

The participants in the two university FGDs discussed how pornography filled a gap in formal sex education. One female participant explained:

*I think that pornography talks about precisely the topics that aren't touched upon in sex education, which is like coitus, like the act, like how to be sensual, how to be desirable, like, the whole part about pleasure […] in school they talk to you about sex as something so scientific, like "ok, so there is penetration, ejaculation and a baby is formed bla bla bla bla". (Female, 19 years, FGD, University)*

This more reflective and positive attitude towards pornography as a source of information on explicit sexual acts contrasted to the dismissive attitude of many younger, particularly female adolescents interviewed.

## Discussion

Two major themes emerging from the findings will be discussed in this paper: the biological and heterosexual focus of sexual health information from trusted sources; and the lack of information on practical skills for ensuring safe and pleasurable sexual experiences.

### Biological reductionism and heteronormativity

Adolescents in this study received considerable information on biological aspects of heterosexual sexuality and reproduction as it related to pregnancy and STIs. Although this is an important part of their sexual health knowledge, the potentially negative effects of this overriding focus on biological and heterosexual sexuality are worth exploring.

When appraising the content of the biologically focussed sexual health information, it becomes clear that adolescents are expected to take rational decisions based on being cognitively aware of negative biological outcomes of unprotected sex. Focussing on biological sexuality without also discussing the emotional/relational aspects of sexuality effectively separates the mind from the body, and the individual from their social context. This artificial separation fails to take into account the emotional and physical impulses that drive sexual attraction, nor does it account for the social forces, gender inequalities and power relations that influence a person's ability to initiate positive health behaviours [28].

To underline this rational decision-making process, fear arousal was a tool utilized by both teachers and parents to warn adolescents about risks and consequences of unprotected sex. However, this information was rarely paired with practical explanations of how to access and use contraception, nor how to negotiate safe sex with a partner. Research has shown that utilization of fear arousal techniques may be counter productive, leading to risk denial *(it won't happen to me)* [29], particularly exacerbated in adolescence by a belief in their own invulnerability or invincibility [1, 3].

This rational perspective also artificially separates the individual from their social context, disregarding the influence of peers, partners and parents on sexual decision-making. In this study negative peer pressure was exemplified though jokes and comments about virginity as well as discrimination of supposedly homosexual peers. This peer pressure may negatively influence sexual health decision making, as found in a South African study where peer pressure undermined health promotion information encouraging safe sex and HIV prevention [30]. Conversely, peers may have a positive influence as seen in this study with the adolescent parents who described their role in promoting safe sex in their peers. When it comes to parents, a study from Cameroon found that perceived parental support for condom use had a strong association with actual condom use [28]. Therefore it seems important that educators promoting safe sex behaviours also include discussions about peer, partner and/or parental attitudes that may greatly influence sexual decision-making.

Rationalization also fails to take into account the influence of conflicting messages from media, in particular pornography and erotic music. In this current study viewing pornography was described as normal for males and attitudes of towards pornography were gendered, consistent with previous research [31]. The sheer extent of adolescent exposure to sexually explicit material on the Internet makes a strong case for broaching the topic of pornography with adolescents to raise their "media literacy" in understanding how media is produced and how to be critical of behaviours promoted through the media [13, 32]. Adolescents in this study were already critical consumers of information on the Internet in general, therefore there is every reason to believe that through critical debate about pornography and other media eroticisation, adolescents can be encouraged to reflect on the conflicting messages between rational risks of unprotected sex and media eroticisation.

Another limitation of biological reductionism is the effect it has on adolescent understandings of sexual orientation. Although there were examples of adolescents whom had learnt about sexual diversity from the perspective of respect and non-discrimination, this approach was far from universal. With an overriding heteronormative, biological focus on prevention of pregnancy in the school setting, the topic of sexual orientation was commonly omitted or explained in biological terms as a hormonal disorder or "condition" making someone different. Additionally, medicalization in the form of referring suspected homosexual, lesbian or bisexual adolescents to psychologists and psychiatrists also supported an understanding of these orientations as conditions in need of medical support. This may further alienate youth with non-heterosexual orientations already at greater risk of poor health outcomes and bullying, whilst failing to explore with these adolescents the emotional and relation aspects of their developing sexuality [33, 34]. Providing support for adolescents in the form of discussing same-sex attraction from a normality perspective focusing on feelings and attractions rather than biological differences may be important in preventing negative social and health outcomes [33, 34]. This support could come from formal sources such as teachers or health professionals. Somewhat paradoxically, through the medicalization of sexual diversity, health professionals in this current study were given a point of contact to provide this emotional support for these youth.

A heteronormative focus may also distort the understanding adolescents have of the wide spectrum of sexual behaviours, regardless of sexual orientation. Adolescents learn about this spectrum of acts primarily from informal sources through conversations with friends and partners, Internet and pornography. In schools, adolescents learnt about the spectrum of sexual acts and particularly non-coital sex through anonymous question sessions. This reflects findings from a study from the United States that analysed the content of anonymous questions written by 795 pupils 11–12 years old and found three times as many questions related to non-coital sex, compared to vaginal sex [35]. This trend indicates that adolescents see their teacher as an appropriate source of this information on diverse sexual acts, however the current taboo of the topic means they only ask in the context of anonymity.

The heteronormative nature of safe sex and abstinence messages were also discussed in the male university focus group where participants observed that many adolescents view oral and anal sex as both physically safe (avoiding pregnancy) and morally safe (maintaining virginity). The idea that oral and anal sex maintain virginity is not unique to the Chilean context, and may be influenced by conservative religious ideologies promoting abstinence and virginity [36]. As these participants discussed, implications of this may include low perception of risk related to anal and oral sex, which could lead to reduced condom use and increased risk of STIs [36]. A further serious implication may be an underreporting of sexual abuse not regarded by adolescents as either sex or abuse.

Another limit to this biological focus was the lack of information on emotional and relation aspects of sexuality in the home and in schools. In the home setting discomfort in talking about *afectividad* was reflected in those parents who provided triggered, short, preventative messages linked to risk of pregnancy and contraception, without developing ideas of love, relationships, feelings, desire or sexual impulses. In schools the limited teaching of *afectividad* in mainstream teaching was linked to the narrow educational and public health agendas focussing on pregnancy and STIs. This is of particular importance in light of results from a Chilean study showing that the main motivations for becoming sexually active described by adolescents are precisely love, emotions and pleasure [17]. Therefore, in order to communicate effectively with adolescents about broad topics of sexuality beyond biology, both parents and teachers may need guidance in confronting their own sexuality, myths, cultural taboos and other barriers to communication on these topics [37].

An interesting emerging finding about school sex education was that themes such as love and what one participant called the "*magic*" of sexuality were often associated directly to religion classes. This raises the concern that non-religious adolescents may dismiss the value of learning about emotional and relational aspects of sexuality in school, if they only associate these teachings with religion. Similarly, by linking the idea of sexual abstinence only to religion, an important part of sex education, namely acknowledging the decision to wait to have sex until after marriage as a valid choice independent of religion, may be dismissed by adolescents and educators alike. In their study from the United States, Jones et al. [8] found that adolescents had a range of definitions of abstinence and that that abstinence messages were often seen as compatible with contraceptive messages. This differs from findings in this current study where abstinence and contraceptive messages were described as mutually exclusive. Therefore, exploring the meaning of abstinence in contemporary Chile presents an interesting area for future research.

Finally, as seen in this study, the narrow public health and educational agendas focused on reducing the number of unplanned pregnancies and STIs meant that sexual pleasure was absent from formal sex education. The only exception was male masturbation, however the complete absence of the topic of female masturbation emphasises a gender disparity that denies female empowerment and entitlement to sexual pleasure [38]. Fine and McClelland [39] criticize the separation of risk and pleasure in formal school sex education, claiming that it distorts the way adolescents understand human relationships and desire. What both male and female adolescents in this current study showed through anonymous questioning in schools, conversations with friends and watching pornography, is that it is very often the topics of pleasure and desire that spark their curiosity.

A focus on the acceptable biological, heteronormative teachings across formal and informal sources is not unique to the Chilean context. Results from quantitative and qualitative studies from countries as varied as Brazil [12], Norway [40], New Zealand [38], Vanuatu [10], the United Kingdom and Australia [37] describe similar situations, suggesting that the heteronormative, biological and risk focus is a tendency in a diversity of global contexts.

### The theory-practice gap

A second emerging theme is the lack of focus on the practical aspects of ensuring optimal sexual health and sexuality. A number of adolescents criticized information from schools and parents for not adequately preparing them with tools to practice safe sex. To underscore the importance learning practical skills, an extensive systematic review of 83 school sex education programs claims that the most effective programs in delaying sexual debut and encouraging protected sex are comprehensive sex education programs that incorporate teaching skills related to partner communication and contraceptive use, as well building self-efficacy to be able to use these skills [4].

Information about contraception was often provided by schools, parents and/or health professionals. Condoms were the primary form of contraception discussed, however the actual mechanism on how to put a condom on was only shown to one adolescent in school. Casas and Ahumada [15] describe how practical teaching about condom use in schools touches on a sensitive nerve in the Chilean context, where there still exists a myth that teaching about contraception, and showing how condoms work will encourage early sexual activity. This myth has been dispelled by extensive evidence showing that comprehensive sex education is more effective than abstinence only sex education in delaying sexual initiation [4, 5].

Another major contraceptive issue raised in this current study was the lack of morally neutral and medically correct information about the emergency contraceptive pill and how to access it. Adolescents had either not received information about the emergency contraceptive, or received vague information as to its efficacy, overshadowed by moralizing messages of guilt and responsibility. Although access is now guaranteed by law in Chile [19], conflicting and ambiguous information about the emergency contraceptive, may impede an adolescent's ability to make an informed decision about its use. Chile is not unique in this respect, as debates on the function and legal status of the emergency contraceptive are common throughout Latin America [41].

A core practical skill for promoting pleasurable and safe sex practices and preventing situations of coercion and miscommunication, is learning how to communicate effectively with a partner about sex [5]. In this study the adolescents overwhelmingly discussed the importance of communicating with a partner on topics of sexual health and sexuality, however actual experiences were varied. These differences may be explained in relation to differing levels of self-efficacy felt by adolescents in power relations with their partners. Breakwell [14] describes the link between the theory of self-efficacy and power, stating that effective communication helps set rules of engagement on when and where sex takes place. Conversely, infrequent partner communication, fear and/or perceived lack of ability to negotiate condom use has been shown to be directly associated with infrequent condom use [42]. This supports the need for an increased focus on communication skills necessary for effective negotiation of consensual, safe and pleasurable sexual experiences.

The final skill relates to setting personal limits and learning to respect other's limits in order to prevent situations of violence and sexual coercion. Few participants in this study reported conversations with trusted adults about setting personal limits and partner violence, representing a gap in sex education. Globally, the majority of gender-based violence is perpetrated by men against women and most of the time this is by someone known to the victim, often their partner [43]. Interestingly, a Chilean study surveying university students found males experienced more dating violence than females, however females were more likely to be physically hurt when exposed to dating violence [44]. Few of these victims had talked with a friend about the violence and none had gone to the police, thus the authors advocate for open discussions with adolescents about partner violence, what it is, how the law can protect them and how to support friends that report violence [44]. Alcohol and drugs may be involved in many coercive sex situations, and although their influence should not be overlooked, focussing only on what potential victims can do to reduce risks may merely perpetuate victim blaming rape myths. Thus it is important to engage both males and females in discussions on issues of respect, consent, personal limits and violence, so they can be respected and empowered in their sexuality. It is a human right to have a sexual life free from coercion, discrimination and abuse, therefore sex education that includes building skills for both setting and respecting personal limits represents a great opportunity to help break the current patterns of sexual violence in Chile.

### Limitations

This study has limitations. Firstly, the voluntary nature of participation means that the sample may be skewed towards adolescents with a strong interest in the topic, or adolescents with particularly open communication with their social network about sexuality issues. The need for parental consent may have bias the sample towards adolescents with open communication with their parents, however three participants whom reported no communication with their parents on sexual health issues still received consent to participate. Finally, there may have been recall bias since participants were asked about experiences they might have had many years earlier. Follow-up interviews and participant validation of the data may have reduced this recall bias, however this was not feasible within the constraints of sampling school pupils.

## Conclusion

Chile has made considerable advances on the sexual and reproductive rights agenda in recent years. Findings in this study suggest that the traditional taboos restricting dialogue about topics such as sexual diversity and abortion seem to be broken and certain prescribed gender roles may be changing, whilst challenges to discussing topics like emergency contraception, sexual violence and sexual pleasure still remain. Therefore it seems an opportune moment to re-evaluate the way in which trusted adults engage with adolescents on these topics. Parents, teachers and health professionals were the most trusted sources of information in this setting, therefore it seems logical to focus interventions on these channels.

In order to promote critical thinking, trusted adults are encouraged to build on the foundation of biological sexual health information currently provided and guide adolescents in reflection on broader topics of sexuality. These topics include sociocultural and media influences on sexual decision-making; sexual orientation, gender and the diversity of sexual practices; peer pressure, discrimination and violence; and emotional and relational aspects of sexuality. Furthermore, it is important to promote development of practical skills in relation to contraceptive use, partner communication and personal limits, as well as building self-esteem and self-efficacy necessary to use these skills. Moving away from fear-based, moralizing approaches towards more reflective and participatory dialogue with adolescents represents a positive step on the way to encouraging adolescent to be critical thinkers and empowered in the decisions they make regarding their sexuality.

## Abbreviations

STI: Sexually transmitted infection
HIV: Human immunodeficiency virus
FGD: Focus group discussion

## Footnotes

1 Information provided by school psychologists and school websites.
